# Newborn Screening for Six Primary Conditions in a Clinical Setting in Morocco

**DOI:** 10.3390/ijns10040080

**Published:** 2024-12-04

**Authors:** Sara El Janahi, Mounir Filali, Zakia Boudar, Amina Akhattab, Rachid El Jaoudi, Najib Al Idrissi, Nouzha Dini, Chakib Nejjari, Raquel Yahyaoui, Michele A. Lloyd-Puryear, Hassan Ghazal

**Affiliations:** 1 Laboratory of Genomic, Epigenetics, Precision and Predictive Medicine, School of Medicine, Mohammed VI University of Sciences and Health, Casablanca 82403, Morocco; seljanahi@um6ss.ma (S.E.J.); zakia.boudar@gmail.com (Z.B.); amina.akhattab45@gmail.com (A.A.); nalidrissi@um6ss.ma (N.A.I.); 2 G Lab Laboratory, Casablanca 20360, Morocco; mounirfilali@glab.ma; 3 Laboratory of Medical Biotechnology, School of Medicine & Pharmacy, University Mohammed V, Rabat 6203, Morocco; r.eljaoudi@um5r.ac.ma; 4 Department of Surgery, School of Medicine, Mohammed VI University of Sciences and Health, Casablanca 82403, Morocco; 5 Pediatric Department, Sheikh Khalifa Ibn Zayed International University Hospital, Mohammed VI University of Sciences and Health, Casablanca 82403, Morocco; nezhadini@gmail.com; 6 Lab of Immunology, Infectious Disease and Tropical Biotechnology, School of Pharmacy, Mohammed VI University of Sciences and Health, Casablanca 82403, Morocco; 7 Euromed Research Center, Euromed University of Fes, Fez 51, Morocco; c.nejjari@ueuromed.org; 8 Faculty of Medicine, Pharmacy, and Dentistry, Sidi Mohamed Ben Abdellah University, Fez 1893, Morocco; 9 Newborn Screening Laboratory. Hospital Regional Universitario de Málaga, IBIMA-Plataforma BIONAND, 29011 Malaga, Spain; raquelyahyaoui@gmail.com; 10 Retired, Senior Scientific Advisor, National Institute of Child Health and Human Development, NIH, Bethesda, ML 20814, USA; mapuryear@gmail.com; 11 Department of Sports Sciences, Royal Institute for Managerial Training in Youth and Sport, Sale 10102, Morocco

**Keywords:** newborn screening, congenital hypothyroidism, congenital adrenal hyperplasia, cystic fibrosis, G6PD deficiency, phenylketonuria, hemoglobinopathies, sickle cell disease, Morocco

## Abstract

Newborn screening (NBS) represents an important public health measure for the early detection of specified disorders; such screening can prevent disability and death, not only from metabolic disorders but also from endocrine, hematologic, immune, and cardiac disorders. Screening for critical congenital conditions affecting newborns’ health is a great challenge, especially in developing countries such as Morocco, where NBS program infrastructure is lacking. In addition, the consanguinity rate is high in Morocco. This study aimed to demonstrate the feasibility of integrating NBS into a diagnostic laboratory for routine analysis. Six primary severe conditions were included: congenital hypothyroidism (CH), cystic fibrosis (CF), phenylketonuria (PKU), glucose-6-phosphate dehydrogenase deficiency (G6PD), congenital adrenal hyperplasia (CAH), and hemoglobinopathies. Methods: A retrospective investigation was carried out to examine the outcomes of NBS in Casablanca, Morocco. A total of 5511 newborn blood samples were collected via heel-prick sampling and tested for the above disorders. Most of the samples were collected within the third and sixth days of birth. The dried blood spots were analyzed via a quantitative immunofluorescence technique and isoelectric focusing. Results: A total of 72 newborns had one of the six pathological conditions. The most prevalent disorders were hemoglobinopathies, which were identified in 47 newborns (0.9%), with 29 having HbC carrier status (0.5%), 15 having Hb S carrier status (0.3%), and 3 having an Hb Bart’s carrier profile (0.05%). This was followed by G6PD deficiency, which was found to affect 16 newborns (0.32% of cases). CF was found in one case (0.02%), whereas five newborns (0.09%) tested positive for CAH. Additionally, two newborns (0.04%) tested positive for CH, and one newborn tested positive for PKU (0.02%). Conclusion: Our findings underscore the importance and success of NBS programs in preventing morbidity and mortality and improving the quality of life of affected neonates. The significant gap in data and research on these disorders within the Moroccan population highlights the urgent need to integrate NBS into routine practice in diagnostic laboratories across Morocco. This integration is crucial for enhancing the health and well-being of Moroccan newborns.

## 1. Introduction

Newborn screening (NBS) represents a significant achievement in public health, involving various tests performed in the first days of a newborn’s life. NBS has significant health consequences and requires rapid and efficient communication between healthcare professionals and their families. NBS aims to identify apparently healthy infants with medical conditions that, if left untreated, could lead to severe health problems, including mental and/or physical disabilities or death [1,2,3]. Although the prevalence of some of these conditions is extremely rare in newborns, the absence of detection and treatment can negatively impact healthy development [3].

The inception of NBS dates back to 1960, when programs initially screened for PKU [4,5]. Since that time, NBS has evolved from a relatively simple single test allowing the detection of a single congenital disorder to become the most successful public health program. While the traditional Wilson and Jungner principles have guided the selection of disorders suitable for public screening [6], the progression of innovative screening methods and novel therapeutic approaches has significantly contributed to the expansion of the spectrum of screened disorders [7]. Mass spectrometry has allowed the detection of more than 50 different conditions [8,9], preventing disability and death not only from metabolic and endocrine disorders but also complementing the screening of hematologic, immune, cardiac, and pulmonary disorders [10,11]. The efforts of the Moroccan Ministry of Health (MMoH) concern the consolidation of mothers’ and infants’ health in the program entitled “Health 2025”, whose objectives are the generalization of the NBS program and the management of certain diseases across the entire Moroccan territory.

The first Moroccan pilot study for NBS included CH screening, which was conducted in eight health centers in Rabat in 1996. This study combined CH screening with the analysis of thyroid-stimulating hormone (TSH) and thyroxine (T4) from dried blood spots (DBS) [12]. Sixteen years later, a collaboration between the Moroccan Ministry of Health (MMoH) and the Japanese International Cooperation Agency (JICA) established an NBS program for CH in public health care clinics in Rabat [13]. In 2019, a Moroccan pilot study on congenital heart disease (CHD) screening pulse oximetry was established in Marrakech in partnership with Children’s National Hospital in Washington, DC, USA [14].

As a lower-middle-income country, Morocco faces significant challenges in establishing an NBS program. Financial limitations, particularly in the public sector, impede the development and implementation of a comprehensive nationwide screening program and the provision of necessary follow-up care and treatment for affected newborns. NBS is not recognized by private healthcare insurance; therefore, screening fees are not covered. Insufficient awareness among healthcare professionals and the general population, largely due to the infrequent prescription of NBS by pediatricians, also hampers the program’s effectiveness. Additionally, inadequate logistical resources and the absence of a national registry for genetic, endocrine, and metabolic disorders prevents accurate estimation of disease prevalence, which in turn impedes the identification of the most common disorders within the Moroccan population from being included in the NBS program.

In Morocco, consanguineous marriages remain prevalent. A study assessing the consanguinity rate among 852 families with a child affected by Down syndrome reported that consanguinity accounted for 15.25% of these cases [13,14]. The study also examined the prevalence of consanguineous marriages among 176 Moroccan couples with a child affected by an autosomal recessive disorder, revealing that 59.09% of these couples were consanguineous [15]. Statistics indicate a birth rate of approximately 17.1/1000, with an annual birth count of approximately 639,201. Thousands of undiagnosed neonates might be at risk of significant mortality or morbidity. Hence, there is a crucial need to institute a nationwide NBS program in the country.

This study aimed to provide an overview of the prevalence of the screened disorders and to assess the feasibility of implementing NBS in diagnostic laboratories to accelerate and facilitate the expansion of screening to various regions of Morocco. Six conditions were included in the NBS program: CH, CF, PKU, G6PDd, CAH, and hemoglobinopathies.

CH is the most common endocrine disorder, with an estimated incidence ranging from 1 in 1138 live births to 1 in 1613 live births in Morocco [12,13]. CF is an inherited autosomal recessive disorder; a Moroccan study estimated that its incidence ranges from 1 in 1680 to 1 in 4150 [16]. While PKU is the most common inherited metabolic disorder of amino acids, unfortunately, to date, no data on the estimated incidence of PKU within the Moroccan population are currently available. G6PDd is an X-linked enzymatic disorder that predominantly affects populations in Africa, Asia, the Middle East, and the Mediterranean [17]. A previous study reported a prevalence of 2.6% among the Moroccan population [18]. CAH is an inherited autosomal recessive disorder with an estimated incidence ranging from 1 in 5000 to 1 in 7000 newborns in Morocco [19]. Hemoglobinopathies encompass all inherited genetic diseases of hemoglobin and are among the most commonly inherited disorders in Africa, Asia, and Mediterranean countries. They include defective synthesis of globin chains (α and β), causing thalassemia syndromes, and mutations resulting in hemoglobin structure variants (e.g., hemoglobin C, E, and S, which cause sickle cell disease (SCD)) [20,21]. According to the World Health Organization (WHO), the prevalence of hemoglobinopathy carriers in Morocco is estimated to be 6.5% [22]. These data reflect a significant gap in data and research on these disorders, suggesting that the Moroccan population may be at significant risk for these six disorders, which need to be explored further.

## 2. Materials and Methods

### 2.1. Study Population

A retrospective study was conducted at the Center of Medical Biology G Lab in Casablanca, Morocco, from 2016 to 2023. A total of 5511 blood samples from full-term newborns were tested. The samples were received from various health institutions, including private clinics and public hospitals, directly from families, and outsourcing laboratories from Casablanca and other cities in Morocco.

### 2.2. Sample Collection

A few drops of capillary blood from neonates were collected on Whatman 903 filter paper through heel-prick using a single-use lancet [4]. Most DBS samples were taken between the 3rd and 6th days of life [23]. The quality and integrity of the samples were inspected, which is essential for accurate testing. DBS should not be compromised by contamination from alcohol used during disinfection or by anticoagulant substances, such as heparin or ethylenediaminetetraacetic acid (EDTA) tubes, and the blood was spotted on filter paper cards and allowed to air dry completely. The cards were subsequently stored at +4 °C [24,25] until analysis to ensure the stability of the analytes and enzymatic activity [26,27].

### 2.3. Sample Analysis Assessment

The DBS were punched via a DBS puncher and analyzed for six primary severe conditions, namely, CH, CF, and 17-OHP, via quantitative assessment via fluorescence immunoassays on a VICTOR2™ D analyzer from Revvity (Turku, Finland), whereas PKU and G6PDd were analyzed via fluorescent chemical assays (ninhydrin method and oxidation test, respectively) via a VICTOR2™ D analyzer, and hemoglobinopathies were assessed via isoelectric focusing. Each parameter was analyzed by the appropriate neonatal screening kit following the manufacturer’s instructions (neonatal screening kit by Revvity; Turku, Finland).

Quality controls provided by the neonatal screening kit were run for each assay to ensure the validity of the results. The data were analyzed according to the Levey Jennings chart. The assay results were accepted only if the control values were within the specified range. To verify the accuracy of the results, the G Lab has enrolled in the US CDC’s Newborn Screening Quality Assurance Program (NSQAP) as part of the preparation for NBS accreditation.

Neonates with initial abnormal or outline screening results underwent repeat testing. For neonates with two confirmed positive screening results, including outlying results, the results were transmitted urgently to the pediatrician. Clinical features and family history were questioned to correlate with the results of the screening. The physician decided on the process of treatment that the newborn would undertake and if supplementary analysis was needed to confirm the results. Alternatively, on the basis of clinical symptoms, screen-positive newborns may start treatment. In some positive cases, the results were confirmed by additional diagnostic analyses as prescribed by the pediatrician. These confirmations included the analysis of specific metabolites and hormones in the serum, as well as molecular tests for 5 known mutations in the G6PD gene and 43 variants in the CFTR gene using the Luminax xTAG CFTR panel of 39 mutations kit (adapted for Caucasians, Hispanic Americans, and Ashkenazi Jewish population). For all our positive-screened infants, follow-up is conducted periodically with their physicians, and treatment is the financial responsibility of the patient’s family. Unfortunately, only the public National Health Institute in Rabat provided follow-up data for screen-positive patients. However, two Moroccan associations, SOS PKU and the “Moroccan Association of Cystic fibrosis”, are working to provide treatment and dietary support for affected patients. The details of the screening tests and recommended confirmatory tests are described in Table 1. 

## 3. Results

This study aimed to assess screening outcomes for six conditions, CH, CF, PKU, G6PDd, 17-OHP, and hemoglobinopathies, in Morocco. Among the 5511 samples from full-term newborns enrolled, 42% were received from private laboratories in various cities across the country, including Tangier, Agadir, Marrakech, Casablanca, Mohammedia, Rabat, Témara, Fes, and Oujda. Thirty percent of the samples were sourced from the neonatal services of private clinics in Casablanca. An additional 21% of the samples were acquired through several pediatricians in Casablanca, who routinely incorporate NBS into their standard neonatal check-ups. Families are referred to local laboratories for specimen collection, which are subsequently sent to the G Lab for analysis; 5% of individuals recognized the importance of NBS or had previously screened their first child, and only 2% were from public health centers (Table 2).

**Figure 1:**
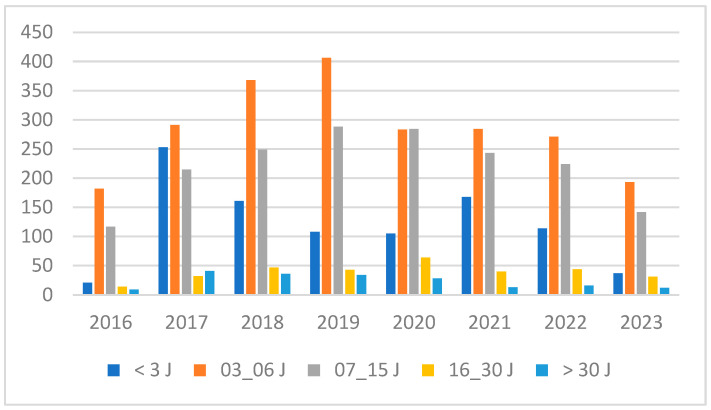
Age range of newborns at DBS sampling.

The samples were collected and tested between 2016 and 2023, with collection ages categorized into five distinct ranges (Table 3). Approximately 41% of the samples were collected between the third and sixth days of life, whereas 32% were collected between the sixth and fifteenth days. A total of 18% of the samples were collected within 24–48 h of birth, with 6% collected between fifteen and thirty days. Only 3% of the samples were obtained after thirty days. Over the years, there has been consistent adherence to the recommended age ranges for sampling and screening (Figure 1). In 2017, 95% of the samples received within twenty-four hours were from clinics, where the sampling occurred before the mother and her newborn were discharged. After gynecologists were sensitized to the importance of timely testing, the samples were consistently collected on the third day of life.

A total of 79 samples from outsourcing laboratories were not screened for all six conditions (Table 4). The primary reasons included insufficient samples or the absence of a second sample to control for positive screening. Among these, there were 45 positive-screened samples for which no confirmatory second sampling was obtained, and there were 23 DBSs with insufficient sample volume. Additionally, 11 newborn DBSs were affected by an anticoagulant due to the use of heparin tubes or EDTA tubes instead of the standard heel-prick method during the sampling process. The use of heparin tubes prevents blood coagulation and ensures that enough sample is placed on the filter paper. However, the use of heparin can potentially affect the accuracy of CAH screening, leading to false-positive results. Despite multiple follow-up attempts with outsourcing laboratories, no second samples were obtained. Notably, we have received several DBSs directly from families with insufficient samples due to challenges at sampling, which prevented us from screening for all the disorders. Therefore, screening was prioritized for the most common and severe conditions as recommended by all the prescribing physicians: CH, CF, and PKU.

Among the 5511 newborn DBS samples, 5400 underwent screening for all six disorders, with specific breakdowns as follows: 5424 samples for CH, 5307 samples for CF, 5429 samples for PKU, 5407 samples for G6PDd, 5400 samples for CAH, and 5411 samples for hemoglobinopathies, as detailed in Table 5.

The NBS study successfully identified a total of 72 positive cases for the six severe conditions, as detailed in Table 5. These results reveal a range of frequencies for the screened disorders. Hemoglobinopathies were the most prevalent, with a rate of 47 cases per 5411 newborns, followed by G6PDd, with 16 cases per 5407 newborns; CF accounted for 1 case per 5307 of the screened population; CAH represented 5 cases per 5405 newborns. CH occurred at a rate of 2 cases per 5424 newborns, and PKU was found at a rate of 1 case per 5429 newborns.

The details of the NBS outcome profiles for the six screened conditions presented below.

Congenital hypothyroidism (CH): A total of 5424 newborns were screened, and 2 newborns tested positive for CH, representing a prevalence of 0.04% live births.

Cystic fibrosis (CF): A total of 5307 newborns were screened, of whom 1 newborn tested positive for CF, with a prevalence of 0.02%.

Phenylketonuria (PKU): In this study, 5429 newborns were screened, and only 1 case presented a value of 8.68 mg/dL, reflecting a prevalence rate of 0.02%.

G6PD deficiency (G6PDd): A total of 5407 newborns were screened, and 16 babies tested positive for this deficiency. This study revealed that G6PD deficiency was the second most common disorder among the six conditions screened, with a prevalence of 0.32% live births.

Congenital adrenal hyperplasia (CAH): Among the 5405 newborns screened for CAH, 5 tested positive. Three of the positive newborns had serum 17 OHP level analysis performed in parallel with the screening test that confirmed the screening. One newborn presented with clinical manifestations of CAH, which were confirmed through screening, whereas another newborn underwent karyotype analysis, confirming the effect. This study revealed a prevalence rate of 0.02% among live births. Notably, 11 newborns with high levels of 17-OHP were found to have false-positive results, likely due to the influence of heparin.

Hemoglobinopathies: By analyzing the isoelectric focusing of hemoglobin profiles, 47 abnormal profiles were identified for variant hemoglobin, with a total prevalence of 0.9%. The 47 cases were as follows: 29 newborns had a profile of HbC carrier status, and 15 presented a profile of HbS carrier status, with 2 of them having their heterozygote state confirmed through hemoglobin electrophoresis. Additionally, three newborns had Hb Bart’s carrier status.

## 4. Discussion

In most African countries, the establishment of national NBS programs continues to be challenging due to various factors, including financial constraints, a lack of awareness, and inadequate logistical resources [17]. There is currently a significant effort to establish sustainable national NBS programs in many developing countries, including Morocco. This NBS retrospective study of six primary conditions, including CH, CF, PKU, G6PDd, 17-OHP, and hemoglobinopathies, within the Moroccan population was performed with the aim of assessing the efficacy of a local NBS program.

Among the 5511 newborns, 5424 who were asymptomatic were screened for CH, and 2/5424 (0.04%) tested positive. This prevalence is lower than the results of the first Moroccan public pilot study for CH screening, which was conducted in eight health centers in Rabat in 1996 and reported a CH incidence of 1 in 1138 newborns [12]. Sixteen years later, a collaboration between the Moroccan Ministry of Health (MMoH) and the Japanese International Cooperation Agency (JICA) established a national NBS program for CHs at public health care sites and estimated a CH incidence of 1/1613 live births in Rabat [13]. Comparatively, a study in the Middle East conducted in Lebanon reported a CH incidence of 1 in 1863 newborns among a total of 408,000 newborns screened [28]. Among the United Arabs of Emirates, out of 1,300,000 screened newborns, 653 tested positive, resulting in a prevalence of 1 in 1991 newborns [28]. In France, all newborns have been systematically subjected to the NBS program for the last 42 years, which revealed a CH prevalence within the French population of 1/2500 newborns [29]. In Portugal, where the national NBS program has been in place for the last 44 years, the CH incidence is 1 in 2892 newborns [30]. These results highlight the higher incidence of CH within the Moroccan population than in European and Middle East countries. The higher prevalence of CH emphasizes the importance of implementing a Moroccan National NBS Program to help with the early identification, diagnosis, and treatment of infants with CH, thereby preventing severe intellectual disability.

Additionally, NBS for CF via the IRT assay allowed the positive screening of one asymptomatic infant who screened positive but did not exhibit any of the identified mutations in the CFTR gene using Luminax xTAG CFTR panel of 39 mutations kit, which indicates the importance of identifying CFTR mutations in the Moroccan population. The prevalence of CF accounts for 1/5307 newborns (0.02%), and this prevalence might be overestimated due to the limited number of samples available for analysis. Currently, comprehensive data on the prevalence of CF in the Moroccan population are lacking. A previous study conducted in the Moroccan population in 2007 tested 150 healthy subjects for 32 CFTR gene mutations and the (T)5 splicing variant of intron 8 to estimate the prevalence of CF mutations in the Moroccan population. The study reported two patients carrying the F508del mutation, and eight subjects were heterozygous for the (T)5 variants. The calculated incidence of CF ranged from 1 in 1680 to 1 in 4150. Therefore, the Moroccan population should be considered at risk of CF and related disorders [16]. In France, the incidence of CF ranges from 1/5321 to 1/4528 newborns [31], closely aligning with the calculated prevalence reported in this study. In Portugal, the prevalence of CF is lower than that in France and Morocco, accounting for 1/9056 newborns. In Middle Eastern countries, the prevalence ranges from 1/2000 to 1/100,000 [32].

The prevalence of PKU varies worldwide, with an average of approximately 1/10,000 newborns [33]. However, to date, we still do not have accurate data on the incidence of PKU in Morocco. In the present study, only one case of PKU was detected, suggesting a PKU incidence of 1/5429 newborns (0.02%). PKU is a condition that may occur more frequently in Morocco, yet it often remains undetected until it reaches a stage of severe intellectual disability. Unfortunately, according to the Moroccan SOS PKU Association, most PKU patients are diagnosed only after symptom onset, at which point, phenylalanine and tyrosine levels are tested to confirm the diagnosis. Efforts to increase awareness about newborn screening through various conferences have resulted in earlier diagnoses of PKU in many newborns and infants. In a study published in 2021, that analyzed phenylalanine concentrations in a cohort of 52 Moroccan PKU patients via tandem mass spectrometry coupled with liquid chromatography, PKU in Morocco was characterized by a predominance of classic and moderate PKU in both sexes. As a result of dietary management, 33 of these patients demonstrated improvements in psychomotor development and a reduction in behavioral disorders [34]. This study highlights that improved patient care might be achievable through the implementation of a comprehensive national NBS program.

According to the World Health Organization (WHO), the prevalence of carriers of hemoglobinopathies in Morocco is estimated to be 6.5% [22]. Hemoglobinopathies have attracted important attention in NBS studies in Morocco [35]. In the present study, 47 abnormal isoelectric focusing hemoglobin profiles were identified. Among these newborns, 29 had HbC carrier status, accounting for 0.5%, 15 had Hb S carrier status, and 2 had a confirmed diagnosis of their heterozygote status via hemoglobin electrophoresis, accounting for 0.3%. Additionally, three newborns had a Hb Bart’s carrier profile, representing 0.05%, whereas, in a study of α-thalassemia prevalence in Morocco, the authors estimated the prevalence of Hb Bart’s carriers in the population to be 1.02% [22], which is higher than the prevalence presented in this study. However, it is the lowest compared with Tunisia (7.38%), Algeria (10%), Tanzania (11.5%), and Congo (14.5%) [22]. In this study, the prevalence of hemoglobinopathies was estimated at 47/5405 newborns (0.9%), which is a very high prevalence that needs further investigation. In a previous retrospective study on sickling hemoglobinopathies conducted in northwestern Morocco, SCD-SS was much more prevalent (81.25%) than was SCD-SC (2.08%) [36]. Between 2015 and 2016, a pilot NBS study, including 1658 newborns, revealed that α-thalassemia has an estimated prevalence of 0.96%, a wide mutation spectrum and a heterogeneous geographical distribution of the disease [22]. The anthropological history of Morocco, marked by its role as a migration crossroads connecting Europe and sub-Saharan Africa, may explain the diversity in the severity of α-thalassemia.

In this study, we estimated the incidence of CAH to be 5/5405 newborns (0.09%). Eleven newborn samples were affected by heparin because of the use of heparin tubes or EDTA tubes instead of the standard heel-prick method during sampling. To address this issue, efforts have been made to contact laboratories and obtain a second compliant sample. However, it is essential to mention that in these instances, the parents did not provide consent for a second sampling. In Morocco, there is no NBS program for CAH; therefore, there are no data on the incidence of this disorder among Moroccan newborns [37]. A study published in 1992 was conducted on 25 Jewish families, of which 19 families were Moroccan and 2 families had one Moroccan parent. The study reported the diagnosis of 38 patients with CAH, 31 of whom were Moroccans, which led to an estimated incidence ranging from 1/5000 to 1/7000 newborns [19]. In France, the prevalence of CAH ranges from 1/15,000 to 1/16,000 newborns [38], highlighting that the prevalence of the disorder within the Moroccan population is twice as high as that in European countries.

G6PDd was the second most common disorder among the six conditions screened in the present study, representing an estimated prevalence of 16/5507 newborns equivalent to 0.3%. Genetic analysis confirmed the diagnoses of G6PDd in three newborns, two of whom were identified as G202A and A376G carriers. Approximately 400 million patients are affected with G6PDd worldwide, mostly affecting African, Asian, Middle Eastern, and Mediterranean countries [17]. These results are lower than those reported in previous Moroccan and Algerian studies (2.6% and 2%, respectively) and significantly lower than the incidence reported in Tunisia and Mauritania (4.4% and 11%, respectively) [18].

The implications of these findings should be considered within the broader context of regional genetic diversity and public health initiatives. Future efforts should focus on promoting the management and follow-up of newborns to increase their quality of life. Further studies should prioritize the identification of Moroccan-specific genetic variants to ensure accurate diagnosis, explore potential genetic and environmental factors to obtain more comprehensive data, and refine our understanding of the prevalence of these conditions within the Moroccan population, which may contribute to the observed differences in incidence rates across North African countries.

## 5. Conclusions

NBS offers an invaluable opportunity to initiate monitoring and treatment at the earliest possible stage, thereby minimizing risks and improving long-term outcomes. This study provides critical insights into the estimated prevalence of several genetic and metabolic disorders, including CH, CF, PKU, G6PDd CAH, and hemoglobinopathies, within the Moroccan population. Among the conditions screened, hemoglobinopathies carrier status was the most prevalent, followed by G6PDd. These results highlight the importance of the NBS program in preventing morbidity and improving the quality of life of affected neonates.

The findings also reveal a significant gap in data and research on these disorders within the Moroccan population, underscoring the urgent need to increase awareness of the importance of NBS, as well as the necessary follow-up and management of affected newborns, to reduce morbidity and improve the health of Morocco’s most vulnerable citizens-neonates. Additionally, the results emphasize the necessity of enhancing research efforts to identify Moroccan-specific genetic variants, which is crucial for accurate diagnosis.

These findings call upon the Moroccan health system to prioritize the recognition of NBS as a routine practice and the establishment of a national registry for diagnosed disorders. Such recognition would support the expansion of the NBS program to include a broader range of critical conditions currently excluded from the Moroccan CH NBS program. This expansion is essential for improving early detection, intervention, and long-term management of these conditions. Furthermore, this paper demonstrates the feasibility of implementing NBS units within diagnostic laboratories, ultimately contributing to the expansion of NBS to all Moroccan cities. The Moroccan NBS program would eventually be based on a public-private partnership as a fundamental strategy for the diagnosis and follow-up management of the neonatal population across the country.

## Figures and Tables

**Table 1:** Details of the screening tests and confirmatory tests recommended for NBS.

Condition Screened	Screening Test	Principle	Units	Reference Range	Recommended Confirmatory Tests
CH	DELFIA neonatal hTSH	Time-resolved Fluoroimmunoassay	uIU/mL	Normal: <10 Borderline: 10–20 Abnormal: >20	Serum TSH, Free T4, Free T3 levels
CAH	DELFIA neonatal 17 alpha-OH-progesterone	Time-resolved Fluoroimmunoassay	nmol/mL	Normal: <17 Borderline: 17–25 Abnormal: >25	Serum 17 OHP levels, cortisol levels, karyotype
G6PD deficiency	Neonatal G6PD	Oxidation test	U/g Hb	Normal: >2.8	G6PD enzymatic activity through spectrophotometry, molecular analysis
Cystic Fibrosis	DELFIA neonatal IRT	Time-resolved fluoroimmunoassay	ug/mL	Normal: <55 Borderline: 55–65 Abnormal: >65	Pancreatitis associated protein (PAP) levels; sweat chloride test; genetic analysis for mutation in *CFTR* gene
PKU	Neonatal phenylalanine	Fluorescent ninhydrin method	mg/dL	Normal: <2.5 Borderline: 2.5–3 Abnormal: >3	Plasma and urine phenylalanine levels; plasma tyrosine levels
Hemoglobinopathies	KIT RESOLVE IEF	Isoelectric focusing		Normal Hb present (FA)	Capillary electrophoresis HPLC

OHP: hydroxyprogesterone; DHEA: dehydroepiandrosterone; ACTH: adrenocorticotropic hormone; TSH: thyroid-stimulating hormone; IRT: immunoreactive trypsinogen; CFTR: cystic fibrosis transmembrane conductance regulator; HPLC: high-performance liquid chromatography; FA Hb: fetal/adult hemoglobin; IEF: isoelectric focusing.

**Table 2:** Origin of newborn blood samples tested.

Origin of Samples	Number of Samples Received	Percentage
Laboratory	2340	42%
Pediatrician	1189	21%
Directly from families	301	5%
Private clinics	1665	30%
Public hospital centers	16	2%

**Table 3:** Age group distribution of DBS samples received.

Age Group	Number of Newborns	Percentage
<3 days	967	18%
3–6 days	2278	41%
7–15 days	1762	32%
16–30 days	315	6%
>30 days	189	3%

**Table 4:** Test distribution of newborn DBS samples unscreened.

Condition Screened	Unscreened Neonates	Insufficient Sample	Inappropriate Sample	Percentage
CH	5	2	-	10%
CAH	12	6	11	37%
G6PD deficiency	11	5	-	24%
CF	3	3	-	9%
PKU	6	3	-	13%
Hemoglobinopathies	8	4	-	18%
Total	45	23	11	

**Table 5:** The results of NBS studies.

Condition Screened	Number of Positive Neonates Screened	Estimated Incidence	Estimated Prevalence
CH	2	2 in 5424	0.4%
CAH	5	5 in 5405	0.09%
G6PD deficiency	16	16 in 5407	0.32%
CF	1	1 in 5307	0.02%
PKU	1	1 in 5429	0.02%
Hemoglobinopathies	47	47 in 5411	0.9%
Total	72		

## Data Availability

All reported data are stored at G Lab laboratory. Due to privacy concerns and participant confidentiality requirements, the data cannot be made publicly available. However, the data can be made available upon request, in full respect of ethical standards.
